# Identification of the SlmA Active Site Responsible for Blocking Bacterial Cytokinetic Ring Assembly over the Chromosome

**DOI:** 10.1371/journal.pgen.1003304

**Published:** 2013-02-14

**Authors:** Hongbaek Cho, Thomas G. Bernhardt

**Affiliations:** Department of Microbiology and Immunobiology, Harvard Medical School, Boston, Massachusetts, United States of America; University of Geneva Medical School, Switzerland

## Abstract

Bacterial cells use chromosome-associated division inhibitors to help coordinate the processes of DNA replication and segregation with cytokinesis. SlmA from *Escherichia coli*, a member of the tetracycline repressor (TetR)–like protein family, is one example of this class of regulator. It blocks the assembly of the bacterial cytokinetic ring by interfering with the polymerization of the tubulin-like FtsZ protein in a manner that is dramatically stimulated upon specific DNA binding. Here we used a combination of molecular genetics and biochemistry to identify the active site of SlmA responsible for disrupting FtsZ polymerization. Interestingly, this site maps to a region of SlmA that in the published DNA–free structure is partially occluded by the DNA-binding domains. In this conformation, the SlmA structure resembles the drug/inducer-bound conformers of other TetR–like proteins, which in the absence of inducer require an inward rotation of their DNA-binding domains to bind successive major grooves on operator DNA. Our results are therefore consistent with a model in which DNA-binding activates SlmA by promoting a rotational movement of the DNA-binding domains that fully exposes the FtsZ-binding sites. SlmA may thus represent a special subclass of TetR–like proteins that have adapted conformational changes normally associated with inducer sensing in order to modulate an interaction with a partner protein. In this case, the adaptation ensures that SlmA only blocks cytokinesis in regions of the cell occupied by the origin-proximal portion of the chromosome where SlmA-binding sites are enriched.

## Author Summary

The tetracycline repressor (TetR)–like protein family is broadly distributed among bacteria. These factors typically regulate the expression of genes coding for drug resistance pumps. When they bind drug molecules, the repressors undergo a conformational change that releases them from their specific DNA-binding sites in order to de-repress pump production. The *Escherichia coli* SlmA protein is a relative of TetR. Instead of regulating gene expression, it blocks cytokinetic ring assembly over chromosomes in order to help coordinate the processes of DNA replication and segregation with cytokinesis. In this report, we identify the active site of SlmA responsible for disrupting the polymerization of FtsZ, a key cytoskeletal protein responsible for organizing the division machinery. Interestingly, the location of the active site on the protein structure indicates that SlmA may represent a special subclass of TetR–like factors that has adapted conformational changes normally associated with drug molecule sensing in order to ensure that SlmA only interacts with FtsZ to block cytokinesis when it is bound to specific sequences of DNA distributed around the chromosome.

## Introduction

Cell division in bacteria typically begins with the assembly of a membrane-associated cytoskeletal structure composed of polymers of the tubulin-like FtsZ protein and its associated binding partners [1]–[5]. This ring-shaped collection of polymers is called the Z-ring and it is ultimately responsible for the recruitment of all known division factors to the prospective site of fission [1]. The maturation of this structure into a functional cytokinetic apparatus appears to take place in two stages [6]. Components of the Z-ring assemble and persist at the division site for about 20% of the cell cycle followed by the recruitment of a large collection of proteins needed to form the active, trans-envelope septal ring machine capable of catalyzing cell constriction [1], [6]. Because it initiates the division process, Z-ring assembly is the ideal target of spatiotemporal regulators directing proper division site selection. Accordingly, in the model organisms *Escherichia coli* and *Bacillus subtilis*, two partially redundant antagonists of Z-ring assembly have been implicated in defining the midcell division plane: the Min system and nucleoid occlusion proteins [7]–[12].

The Min system has been extensively studied and its function is relatively well understood [13], [14]. The FtsZ antagonist MinC is the key output of the system. Its subcellular localization is controlled by additional Min proteins such that it either rapidly oscillates from pole-to-pole as is the case in *E. coli* [15]–[17], or is recruited to both poles in the case of *B. subtilis* [18]–[20]. In both systems, a gradient of MinC is thought to be formed such that its concentration is highest at the cell poles and lowest at midcell, thus making this position the favored location for FtsZ polymerization and Z-ring assembly [13], [21].

Negative control of Z-ring assembly by the nucleoid, a phenomenon referred to as nucleoid occlusion [9], is mediated by chromosome-associated division inhibitors. Several years ago, the nucleoid occlusion factors Noc and SlmA were identified in *B. subtilis* and *E. coli*, respectively [22], [23]. Noc is a member of the ParB family of DNA-binding proteins whereas SlmA is related to TetR. Considering that the two proteins are completely unrelated at the sequence level, they share a striking number of features [11]. In their respective organisms, inactivation of the nucleoid occlusion factor is synthetically lethal with a defect in Min system function, and both Noc and SlmA are required to prevent division over chromosomes when problems with DNA replication/segregation are encountered [22], [23]. SlmA and Noc were also both recently shown to be specific-DNA binding proteins [24], [25]. Although their binding sequences differ, the positions of their binding sites on their respective chromosomes are remarkably similar; they are broadly distributed throughout the origin-proximal 2/3 of the chromosome, but completely absent in the terminus region [24]–[26]. Because sequences near the origin are the first to be replicated and segregated, chromosomally-associated nucleoid occlusion factors are likely to be segregated to opposite daughter cell halves to create an inhibitor-free zone at midcell that allows Z-ring assembly at a time when replication is nearing completion [24]–[26]. While not essential, the timing of nucleoid occlusion factor segregation dictated by their binding site distribution appears to be one of the cellular mechanisms responsible for properly coordinating chromosome replication and segregation with cell division. Consistent with this idea, moving NO factor binding sites to the terminus region of both *E. coli* and *B. subtilis* delays the division process [24], [25].

SlmA has been shown to directly regulate FtsZ assembly [23], [25], [26]. The target of Noc regulation, on the other hand, currently remains unknown. We recently showed that SlmA functions as an antagonist of FtsZ polymerization *in vitro* and that its anti-FtsZ activity is greatly stimulated upon binding to specific SlmA-binding sequences (SBSs) [25]. Importantly, a SlmA variant, SlmA(R73D), found to be defective for FtsZ regulation but not DNA-binding *in vivo* was also defective in interfering with FtsZ polymerization *in vitro*. Thus, the biochemical activity observed for purified SlmA is likely to be physiologically relevant. However, an alternative model for SlmA function has also recently been proposed by Schumacher and colleagues in which dimers of SlmA promote the formation of anti-parallel FtsZ protofilaments, one emanating from each monomer, such that the protofilaments cannot productively contribute to Z-ring formation [26]. This model is based on small-angle X-ray scattering analysis of purified SlmA-FtsZ complexes and the electron microscopic observation that FtsZ forms twisted bundle structures *in vitro* in the presence of SlmA-SBS complexes. The physiological significance of these twisted FtsZ bundles remains unclear [25].

To better understand SlmA activity, we sought to identify additional SlmA variants defective in their ability to antagonize FtsZ assembly. We therefore developed a selection and screen combination to identify *slmA* alleles encoding variants that fail to properly regulate Z-ring assembly but retain DNA binding activity. Several mutants with these characteristics were isolated and the variants they encode contain substitutions that cluster on the SlmA structure at a site removed from the dimerization interface. When purified, several of these SlmA derivatives failed to interact with FtsZ *in vitro*. The affected residues thus identify an FtsZ-interaction site on SlmA. Importantly, this site maps to a region of SlmA that in other TetR-like proteins is conformationally flexible. Structural analysis of several dimeric TetR-like factors indicates that when they bind inducer/drug molecules their DNA-binding domains rotate away from each other [27], [28]. This rotation results in the dissociation of repressor-operator complexes and the derepression of pump production because the spacing of the recognition helices becomes too wide for binding to successive major grooves on DNA. A similar, non-optimal spacing of recognition helices is observed in the published DNA-free structure of SlmA [26]. Interestingly, in this conformation, the anti-FtsZ active site we have identified is partially occluded by the DNA-binding domains. Our results are therefore consistent with a model in which DNA-binding activates SlmA by promoting the inward rotation of its DNA-binding domains to fully expose the FtsZ-binding sites. SlmA may thus represent a special subclass of TetR-like proteins that have adapted conformational changes normally associated with inducer sensing in order to modulate an interaction with a partner protein. In this case, the adaptation ensures that SlmA only blocks cytokinesis in regions of the cell occupied by the origin-proximal portion of the chromosome where SlmA-binding sites are enriched.

The identification of FtsZ-interaction defective SlmA variants also allowed us to test the functionality of obligate heterodimers containing only one operational active site. Our results indicate that SlmA dimers are capable of properly regulating FtsZ-ring assembly when only one of the monomers possesses a functional FtsZ-interaction interface. We therefore infer that the formation of anti-parallel FtsZ polymers is unlikely to be the mechanism by which SlmA regulates cell division.

## Results

### Genetic strategy for the identification of SlmA variants defective for FtsZ regulation

**Figure 1:**
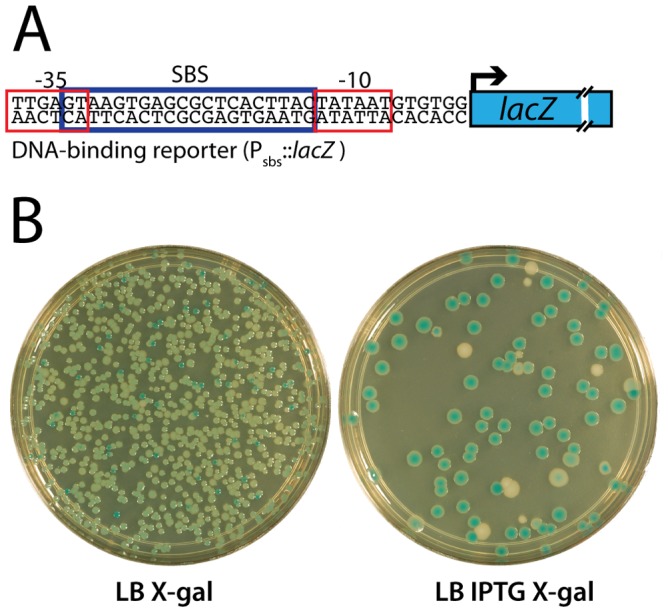
Selection for mutants producing SlmA variants defective in FtsZ regulation. A. Shown is a diagram of the P_sbs_::*lacZ* reporter construct indicating the relative positions of the SBS and promoter elements. The synthetic promoter replaces the *lac* promoter at the native *lac* locus in the chromosome. B. A frozen aliquot of slurried HC328(attHKHC583)/pHC534 [Δ*slmA* P_sbs_::*lacZ* (P_lac-m3_::*slmA*)/pUC-2xSBS] cells with PCR-mutagenized *slmA* was thawed, diluted to 10^−7^, and 100 µl of the dilution was spread on the indicated agar plates. The plates were incubated overnight at 30°C for two days and photographed. As shown, induction of mutagenized *slmA* with IPTG in the parental strain resulted in a plating defect of approximately an order of magnitude.

Functional SlmA induces a lethal division block in cells harboring a multi-copy plasmid encoding tandem SBSs [25]. This phenotype is caused by the global inhibition of Z-ring assembly by plasmid-borne SlmA-SBS complexes dispersed throughout the cell [25]. Therefore, to identify residues in SlmA responsible for its anti-FtsZ activity, we selected for *slmA* alleles that fail to block cell division in cells possessing a multi-copy SBS-containing plasmid. For the selection, the *slmA* gene was subjected to PCR-based mutagenesis and inserted into an integration vector, pHC583, under control of a synthetic lactose promoter (P_lac-m3_). The resulting plasmid library was then integrated at the phage HK022 *att* site of strain HC328 [Δ*slmA* P_sbs_::*lacZ*] in which the native *slmA* gene was deleted. Finally, the pUC-derivative, pHC534, encoding tandem SBSs, was introduced into the library and survivors were selected on LB agar containing 1 mM IPTG to induce *slmA* expression from the integrated construct. In order to rapidly identify *slmA* alleles encoding protein variants specifically defective in FtsZ regulation, we employed a secondary screen to assess the DNA-binding activity of SlmA in the surviving colonies. A *lacZ* reporter was generated (P_sbs_::*lacZ*) with an SBS site between the −10 and −35 elements of the promoter so that *lacZ* expression is repressed upon SlmA binding to the SBS (Figure 1A). With this reporter construct in the selection strain, colony color in the presence of the LacZ substrate X-gal allowed us to visually discriminate survivors that are likely to encode unstable, truncated, or otherwise DNA-binding defective SlmA variants (blue, *lacZ* expressed) from those likely to be defective solely in FtsZ regulation (white, *lacZ* repressed) (Table 1 and Figure 1B). As shown in Figure 1B, the efficiency of plating of the HC328-derived parental strain in the presence of 1 mM IPTG was approximately 10^−1^ following *slmA* mutagenesis. Only a small fraction of the survivors produced SlmA variants that retained DNA-binding activity as indicated by their white color. To eliminate *slmA* alleles encoding proteins with functional but reduced DNA binding activity, white colonies were further purified on LB X-gal plates containing a lower concentration of inducer (100 µM IPTG). With one exception, alleles that behaved like a *slmA*(R73D) control and remained white after several days of incubation (27 of 44 isolates) were chosen for further study.

**Table 1:** Expected colony phenotypes for *slmA* mutants in strain HC328/pHC534 [Δ*slmA* P_sbs_::*lacZ*/pUC-2xSBS].

	Expected Phenotype^a^	
SlmA variant produced	−IPTG	+IPTG
WT	light blue colony	no growth
unstable or truncated	dark blue colony	dark blue colony
DNA-binding defective	dark blue colony	dark blue colony
FtsZ regulation defective	light blue colony	white colony

a For cells plated on LB X-gal supplemented with 1 mM IPTG.

**Figure 2:**
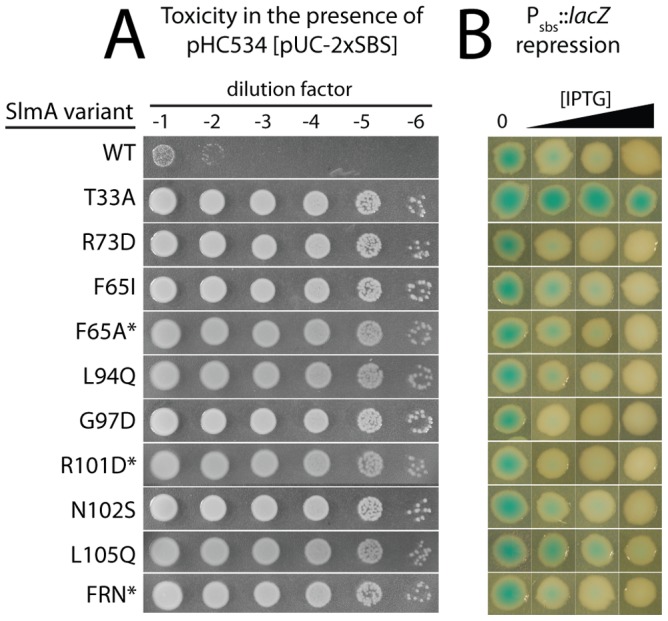
*In vivo* assays for assessing the toxicity and DNA-binding activity of SlmA variants. A. Overnight cultures of HC328/pHC534 [Δ*slmA* P_sbs_::*lacZ*/pUC-2xSBS] cells containing integrated expression constructs (pHC531-derivatives) producing the indicated SlmA variant were serially diluted following normalization for culture OD_600_. Five microliters of each dilution was spotted onto LB agar supplemented with IPTG (1 mM) to induce production of the SlmA variants. Plates were incubated overnight at 30°C and photographed. Note that in order to rescue a Min− SlmA− growth defect, integrated pHC531 constructs and their derivatives must be induced with a minimum of 500 µM IPTG. Thus, using 1 mM IPTG for induction, SlmA is mildly overproduced to about 4X native levels (see Figure 5). This concentration of SlmA has no adverse effect on cell growth or division in the absence of SBS-containing plasmids. B. HC328 cells with integrated pHC531-derivatives but lacking the pHC534 (pUC-2xSBS) plasmid were streaked on LB agar containing 0, 50, 100, or 250 µM IPTG and X-gal. The plates were incubated as above for two days at 30°C and photographed. Representative colonies of each strain were cropped from the plate image and displayed in the same row. IPTG concentrations increase from left to right. Note that mutants marked with an asterisk were made by site-directed mutagenesis following the original genetic selection/screen. FRN refers to a SlmA derivative containing a combination of three substitutions: F65A, R73D, and N102S. See text for details.

**Figure 3:**
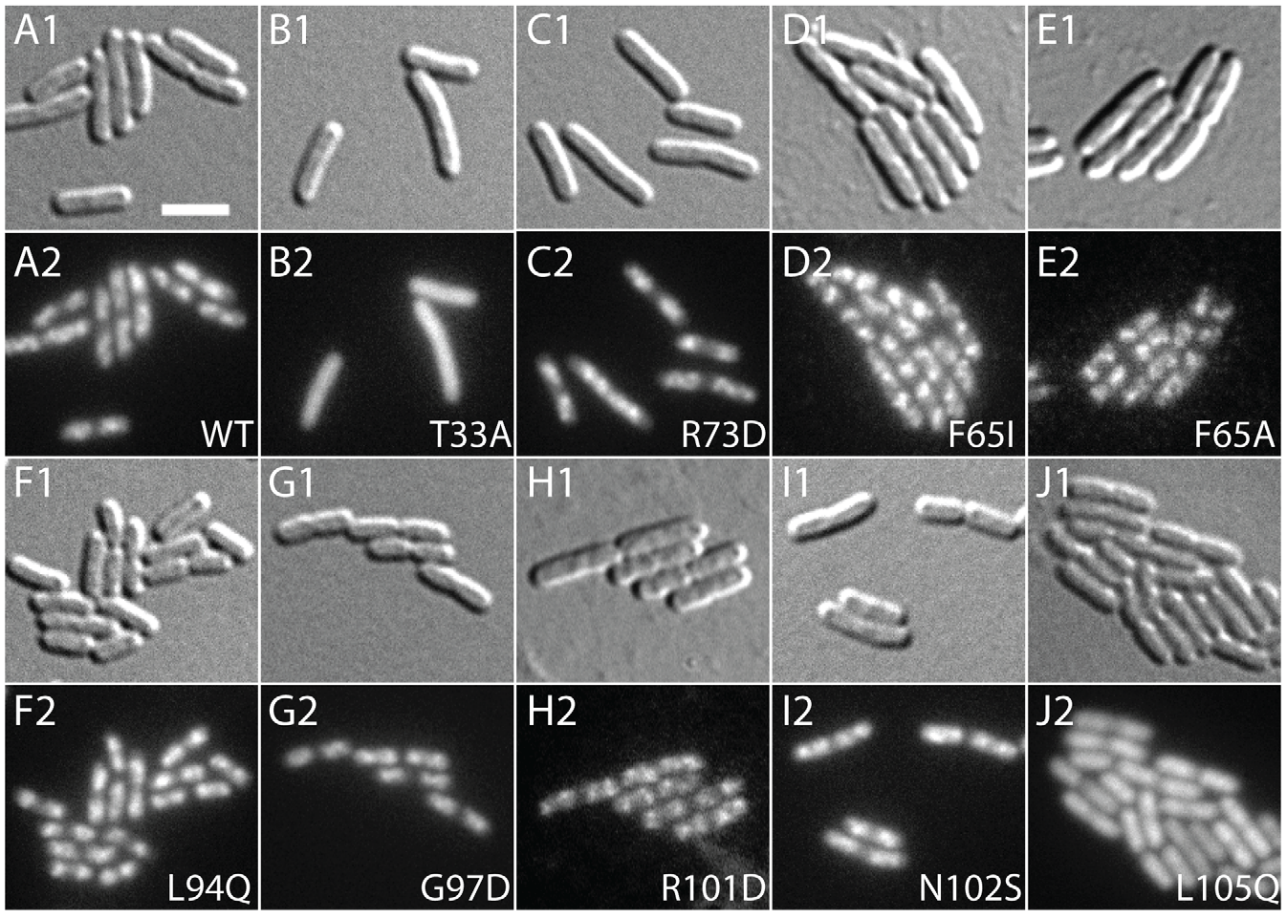
Subcellular localization of GFP-SlmA fusions. Cells of HC259 [Δ*slmA*] with the integrated expression plasmids: pHC625 [P_lac_::*gfp-slmA*(WT)] (A), pHC505 [P_lac_::*gfp-slmA*(T33A)] (B), pHC482 [P_lac_::*gfp-slmA*(R73D)] (C), pHC628 [P_lac_::*gfp-slmA*(F65I)] (D), pHC684 [P_lac_::*gfp-slmA*(F65A)] (E), pHC631 [P_lac_::*gfp-slmA*(L94Q)] (F), pHC629 [P_lac_::*gfp-slmA*(G97D)] (G), pHC685 [P_lac_::*gfp-slmA*(R101D)] (H), pHC627 [P_lac_::*gfp-slmA*(N102S)] (I), or pHC630 [P_lac_::*gfp-slmA*(L105Q)] (J) were grown to an OD_600_ of 0.8–1.0 in LB supplemented with 1 mM IPTG and imaged with DIC (panel 1) and GFP (panel 2) optics. Bar equals 3 microns. Note that as reported previously [23] the GFP-SlmA(WT) signal closely resembles that of nuceloids stained with the DNA-stain DAPI.

The *slmA* gene from the integrated expression construct of each allele was amplified by PCR and sequenced. Many isolates were found to have the same substitution, either alone or in combination with another mutation (Table 2). The most common substitutions observed among the mutant alleles were those in codons 65 and 102 with a minority mapping to codons 94, 97, and 105 (Table 2). The *slmA* genes from mutants possessing a single substitution at these positions were re-cloned into plasmid pHC531 where they were again under P_lac-m3_ control. The resulting constructs were then integrated at *att*λ in the original parental strain HC328 [Δ*slmA* P_sbs_::*lacZ*] for phenotypic analysis. As expected, all of the reconstructed mutant alleles were not toxic when they were expressed in the presence of the multi-copy SBS-containing plasmid pHC534 (Figure 2A). We also assessed the ability of the encoded SlmA variants to repress *lacZ* expression from the P_sbs_::*lacZ* reporter by monitoring colony color development on X-gal plates following induction with a range of IPTG concentrations (50–250 µM) (Figure 2B). The previously characterized variants SlmA(T33A) and SlmA(R73D) served as controls for DNA-binding defective and proficient proteins, respectively [25]. SlmA(T33A) failed to repress P_sbs_::*lacZ* and formed blue colonies at all IPTG levels (Figure 2B). SlmA(R73D), on the other hand, blocked LacZ production and formed white colonies at all inducer concentrations tested (Figure 2B). Most of the newly identified variants were as effective as SlmA(WT) and SlmA(R73D) at repressing P_sbs_::*lacZ*. The exception was SlmA(L105Q), which required 250 µM IPTG for detectible repression. This result was expected because the original isolate yielded a light-blue color when it was originally purified on agar with 100 µM IPTG. Results with the P_sbs_::*lacZ* reporter were perfectly consistent with an analysis of the subcellular localization of each variant. When fused to GFP, all of the variants that repressed *lacZ* expression at low IPTG concentrations were found to have a distinct nucleoid localization pattern that is also indicative of intact DNA-binding activity (Figure 3). GFP-SlmA(L105Q), however, had a more diffuse localization pattern approaching that of GFP-SlmA(T33A), which is almost completely defective for DNA-binding [25] (Figure 3).

**Table 2:** Amino acid substitutions in SlmA variants identified with the selection and screen combination.

substitution	# of isolates	# isolates with a single substitution	toxicity in cells with pHC534 (pUC-2xSBS)	P_sbs_::*lacZ* repression	complementation of Min^−^ SlmA^−^ growth defect
F65L	9	3	−	+	N.D.
F65S	5	2	−	+	N.D.
F65I^a^	1	1	−	+	+/−
F65Y	1	0	−	+	N.D.
L94Q^a^	1	1	−	+	+/−
G97D^a^	2	1	−	+	+/−
N102S^a^	5	4	−	+	−
N102T	1	1	−	+	N.D.
L105Q^a^	2	2	−	+/−	−

a Phenotypes for these substitutions were assessed with reconstructed expression cassettes as well as the original isolates, all others were tested only in the context of the original isolates often in the presence of additional substitutions.

**Figure 4:**
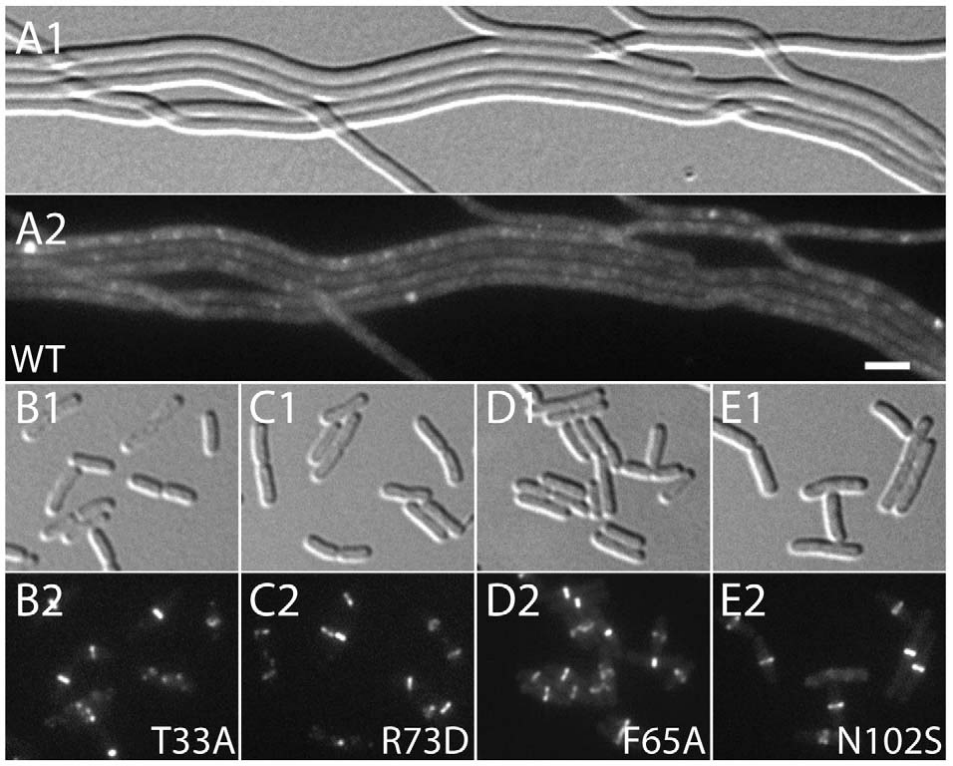
Effect of SlmA variants on Z-ring formation in the presence of multi-copy SBS. Cells of HC290/pHC534 [*zapA-gfp* Δ*slmA*/pUC-2xSBS] with the integrated expression plasmids: pHC531 [P_lac-m3_::*slmA*(WT)] (A), pHC544 [P_lac-m3_::*slmA*(T33A)] (B), pHC543 [P_lac-m3_::*slmA*(R73D)] (C), pHC678 [P_lac-m3_::*slmA*(F65A)] (D), and pHC610 [P_lac-m3_::*slmA*(N102S)] (E), were grown to an OD_600_ of 0.6 in LB supplemented with ampicillin (50 µg/ml) and 1 mM IPTG and imaged with DIC (panel 1) and GFP (panel 2) optics. Bar equals 3 microns. Note that a GFP fusion to the FtsZ-binding protein ZapA [42] is used as a proxy for Z-ring formation and that the ZapA-GFP rings in panels B–E are identical to those observed in wild-type cells.

**Figure 5:**
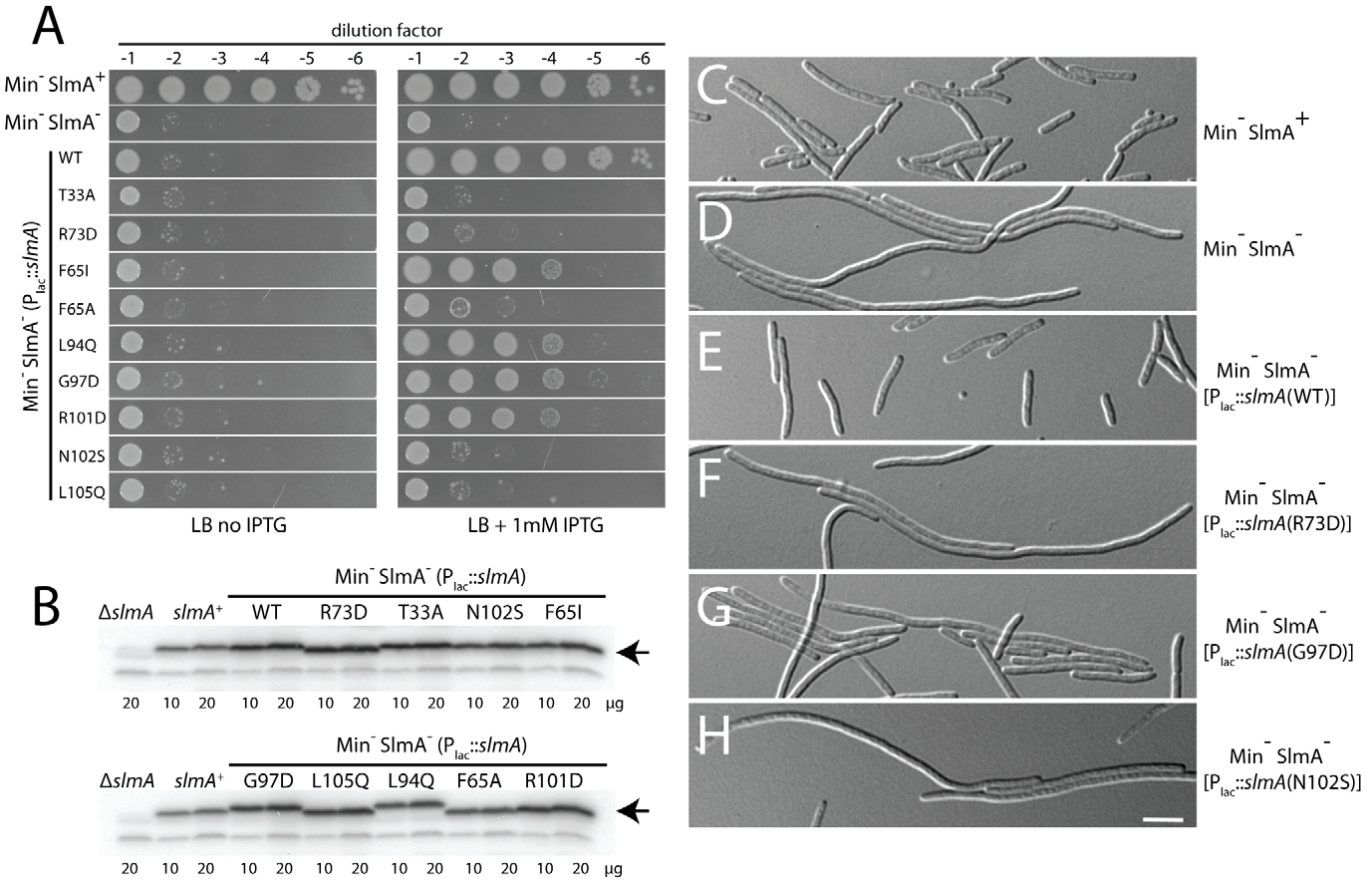
Nucleoid occlusion activity of the SlmA variants. A. Overnight cultures of TB57 [P_ara_::*minCDE*], HC278 [P_ara_::*minCDE* Δ*slmA*], and HC278 containing integrated expression plasmids producing the indicated SlmA variant were diluted and plated on the indicated medium as described in the legend for Figure 2. B–C. The same strains were grown in LB broth supplemented with 1 mM IPTG to an OD_600_ of 0.6. Protein extracts were prepared and proteins in 10 and 20 µg of total extract were separated by SDS-PAGE. SlmA was then detected by immunoblotting with affinity-purified anti-SlmA antibodies (B). The division phenotype of cells from the resulting cultures was also observed using DIC optics. Images for a representative set of strains are shown (C–H). Bar equals 5 microns.

To assess the division regulatory activity of the SlmA variants further, we tested their effect on Z-ring formation in the presence of pHC534 [pUC-2xSBS] and their ability to complement the synthetic lethal phenotype of Min^−^ SlmA^−^ cells. Unlike the SlmA(WT) construct, none of the reconstructed mutant plasmids interfered with Z-ring formation in pHC534-containing cells following induction of the *slmA* alleles (Figure 4). To investigate the native nucleoid occlusion activity of each variant, the *slmA* expression constructs were transduced into HC278, a Δ*slmA* strain in which *minCDE* expression is controlled by the arabinose promoter. We then assayed the ability of the SlmA variants to support growth in the absence of arabinose (Min^−^ SlmA^−^ conditions) (Figure 5A). Even though the variants behaved similarly in other assays and were found to be produced at similar levels (Figure 5B), a range of nucleoid occlusion defects were observed. SlmA(N102S) and SlmA(L105Q) displayed the most severe loss of activity, resulting in plating defects for the HC278 strain that were identical to the previously characterized defective variants SlmA(T33A) and SlmA(R73D) (Figure 5A). All other variants identified in the selection/screen showed an intermediate nucleoid occlusion defect, resulting in a less severe reduction in plating efficiency for the test strain and slow growth of the colonies that formed. The magnitude of the plating defects observed correlated well with the division phenotypes displayed by Min^−^ SlmA^−^ cells producing different SlmA variants (Figure 5C–5H). Cells producing SlmA(N102S) and SlmA(L105Q) primarily formed long non-septate filaments resembling Min^−^ SlmA^−^ cells without a *slmA* expression construct (Figure 5D, 5H, and data not shown). On the other hand, cells producing variants with an intermediate phenotype formed a heterogeneous mix of elongated and filamentous cells indicative of nucleoid occlusion being partially functional (Figure 5G and data not shown). Based on our overall phenotypic analysis, we conclude that our selection/screen combination has successfully identified additional residues in SlmA that are likely to be specifically required for antagonizing Z-ring formation.

### Substitutions in the defective SlmA variants identify a potential FtsZ-interaction interface

**Figure 6:**
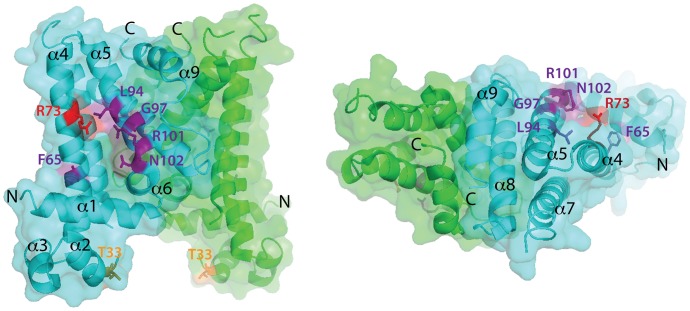
Location of amino acid substitutions on the SlmA structure. Shown are two views of the SlmA dimer structure [26] with one subunit colored cyan and the other green. On the left view, the HTH motif is facing the bottom of the page and on the right it is facing into the page. N- and C- termini as well as helices 1–9 are labeled for reference. The location of SlmA residues identified in this study as being important for FtsZ regulation are highlighted in purple. Residues R73 and T33 for which the effect of substitutions has been previously studied are highlighted in red and orange, respectively. Note that F65 is occluded by the HTH domain.

When mapped onto the SlmA structure [26], the residues identified as being critical for the division regulatory activity of SlmA were found to cluster on two adjacent alpha-helices (α4 and α5) just above the N-terminal helix (α1) that precedes the canonical helix-turn-helix (HTH) motif (α2–α3) (Figure 6). We therefore performed site-directed mutagenesis to create substitutions at neighboring positions (Table 3) with the goal of identifying additional residues important for SlmA function. Using the battery of phenotypic assays described above, we found that an R101D substitution also results in a SlmA protein that is likely to be functional for DNA-binding but defective for division regulation (Figure 2, Figure 3, Figure 4, Figure 5). Additionally, we found that an F65A substitution results in a protein that is more defective for nucleoid occlusion than the F65I variant characterized above (Figure 5A–5B). Overall, the variants with the greatest nucleoid occlusion defects that remained capable of P_sbs_::*lacZ* repression were the previously characterized SlmA(R73D) variant and the newly identified SlmA(F65A) and SlmA(N102S) derivatives (Figure 5A–5B). Combining these substitutions into a single variant SlmA(FRN) also resulted in a protein defective for regulating Z-ring formation that retained P_sbs_::*lacZ* repression activity comparable to SlmA(WT) (Figure 2). Since their activities in the *in vivo* assays fit those expected for variants strongly defective in interacting with FtsZ, SlmA(F65A), SlmA(R101D), SlmA(N102S), and SlmA(FRN) were purified to further study their DNA-binding and anti-FtsZ activities *in vitro*.

**Table 3:** Amino acid substitutions in SlmA generated by site-directed mutagenesis.

substitution	toxicity in cells with pHC534 (pUC-2xSBS)	P_sbs_::*lacZ* repression	complementation of Min^−^ SlmA^−^ growth defect
F65A	−	+	−
F65Y	+	N.D.	N.D.
S69A	+	N.D.	N.D.
S69N	+	N.D.	N.D.
R101A	+	N.D.	N.D.
R101D	−	+	+/−
L105M	+	N.D.	N.D.

### *In vitro* activity of the SlmA variants

**Figure 7:**
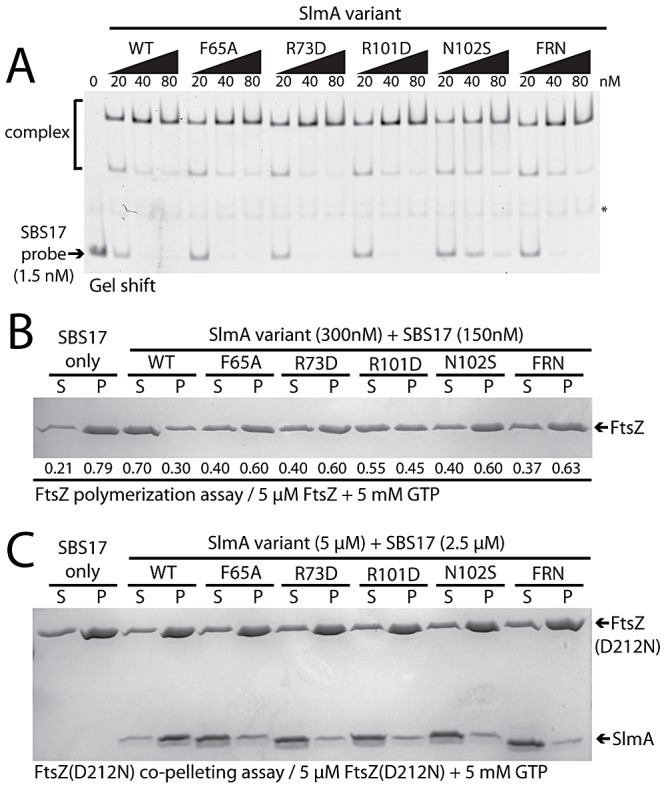
*In vitro* characterization of SlmA variants. A. Gel-shift analysis of SlmA DNA-binding activity. SlmA(WT) or the indicated variants (20–80 nM) were incubated with a Alexa488-labeled SBS17-containing DNA fragment (105 bp, 1.5 nM) prepared by PCR with an end-labeled primer. Protein-DNA complexes were resolved on a 6% polyacrylamide gel and DNA was visualized using a Typhoon fluorescence imager. The light-band marked with an asterisk is a non-specific PCR product formed during probe preparation. B. FtsZ pelleting assays. FtsZ (5 µM) was incubated with GTP (5 mM), SBS17 DNA (150 nM), and the indicated SlmA derivative (300 nM) at room temperature for 15 min. The reactions were centrifuged at 80,000 rpm in a TLA100.2 rotor for 15 min in a tabletop ultracentrifuge and and proteins in the supernatant and pellet fraction were separated on an 12% SDS-PAGE gel and stained with coomassie brilliant blue. Numbers under the bands correspond to the fraction of total FtsZ present in the supernatant/pellet. C. FtsZ(D212N) (5 µM) was incubated as above with GTP (5 mM), SBS17 DNA (2.5 µM), and the indicated SlmA derivative (5 µM). Reactions were then centrifuged and processed as described above.

As expected from the P_sbs_::*lacZ* repression results, gel-shift analysis using a 105 bp probe containing the chromosomal SBS17 sequence [25] indicated that all of the purified SlmA variants retained near normal DNA-binding activity (Figure 7A). The only variant displaying a minor reduction in DNA-affinity was SlmA(N102S). Conversely, all of the variants tested showed a defect in antagonizing FtsZ polymerization in the presence of SBS17 DNA as assessed using an FtsZ pelleting assay [29] (Figure 7B). Importantly, the magnitude of the biochemical defect appeared to correlate nicely with the nucleoid occlusion defect observed for the mutants *in vivo*. For example, SlmA(F65A) showed a relatively robust defect in its ability to block FtsZ polymerization *in vitro* (Figure 7B), comparable to that of SlmA(R73D), and was found to be largely incapable of supporting growth of Min^−^ SlmA^−^ cells (Figure 5A). SlmA(R101D), on the other hand, was able to partially restore growth to a Min^−^ SlmA^−^ strain when it was moderately overproduced (Figure 5A–5B). Accordingly, this variant displayed an intermediate defect in its anti-FtsZ activity *in vitro* (Figure 7B).

To determine if the inability to properly interfere with FtsZ polymerization was the result of a defect in FtsZ-association, we tested whether or not the purified SlmA variants could bind FtsZ(D212N). This FtsZ derivative is defective for GTPase activity, and we previously used it to show that functional GTPase activity is required for SlmA to antagonize FtsZ polymerization [25]. Instead of breaking down polymers of FtsZ(D212N), SlmA-SBS complexes stably associate with the protofilaments and can be co-pelleted with them. All of the new SlmA variants tested failed to pellet with FtsZ(D212N) polymers (Figure 7C), indicating that they are incapable of binding to protofilaments. We therefore conclude that the substituted residues identified using our genetic approach define a critical FtsZ interaction interface on the surface of SlmA (Figure 6).

### Only one FtsZ-interaction interface per dimer is required for SlmA function

According to the model of Schumacher and colleagues, dimers of SlmA disrupt Z-ring assembly by promoting the formation of antiparallel FtsZ protofilaments. Each monomer of the SlmA dimer is thought to bind one of the differentially oriented protofilaments to promote the disruptive anti-parallel configuration. This model predicts that mixed dimers composed of a WT monomer and one with a defective FtsZ-interaction interface should fail to properly regulate Z-ring assembly because they cannot generate antiparallel FtsZ protofilaments. To test this prediction, SlmA variants that can only function as heterodimers were required.

**Figure 8:**
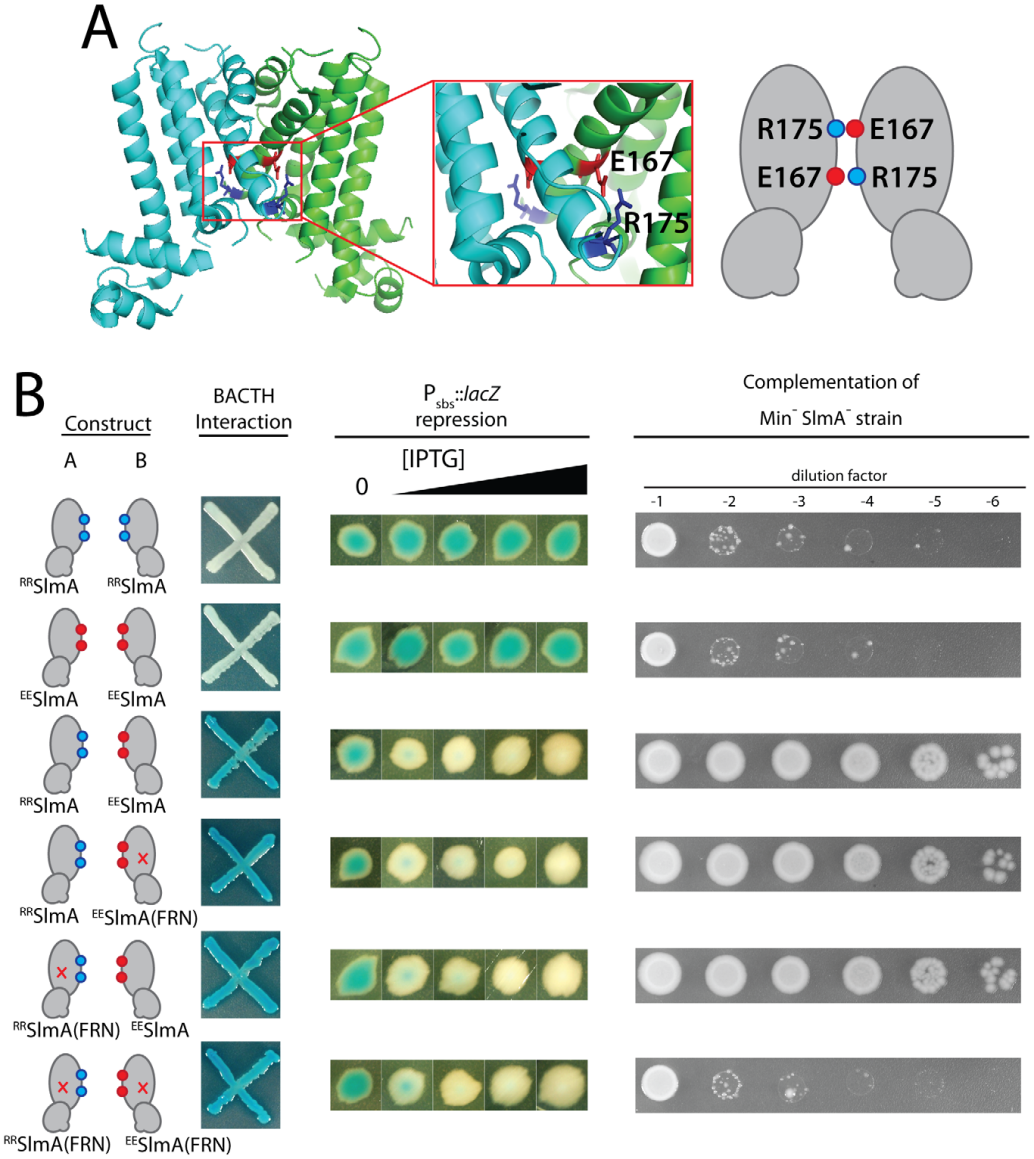
Activity of obligate SlmA heterodimers. A. Shown is the structure of SlmA highlighting the potential electrostatic interaction between E167 and R175 across the dimer interface. A cartoon representation of the interaction is given to the right of the structure. B. SlmA charge-swapped variants, ^RR^SlmA and ^EE^SlmA, were produced from compatible pairs of constructs labeled A and B. For the BACTH analysis, fusions of the indicated SlmA variant to T25 were produced from construct A, and fusions to T18 were produced from construct B. All combinations of T25 and T18 fusions were tested, but only a subset are shown (see text for details). Plasmid pairs encoding the fusion proteins were co-transformed into BTH101 [*cya*-99]. Individual colonies were patched on M9-glucose supplemented with Amp, Kan, X-gal, and 250 µM IPTG. Plates were incubated at 30°C and photographed after 30 hours. For this particular BACTH assay, interacting partners bring together T18 and T25 to reconstitute adenylate cyclase activity [30]. This is detected using *lacZ* induction as a reporter, and blue color is indicative of a positive interaction. For the tests of P_SBS_::*lacZ* repression and rescue of a Min^−^ SlmA^−^ growth defect, SlmA variants were under control of the P_lac-m3_ promoter. Variants listed in column A were produced from an expression construct integrated at *att*λ, and variants listed in column B were produced from an expression construct integrated at *att*HK022. Analysis of P_SBS_::*lacZ* repression was performed as described in Figure 2B. For testing nucleoid occlusion activity, overnight cultures of strain HC278 [P_ara_::*minCDE* Δ*slmA*] containing integrated expression plasmids producing the indicated SlmA variants were diluted and plated on LB medium supplemented with 500 µM IPTG as described in the legend for Figure 2.

Inspection of the SlmA structure [26] revealed a potential electrostatic interaction between residues E167 and R175 across the dimer interface (Figure 8A). We therefore reasoned that derivatives with an E167R substitution (^RR^SlmA) or an R175E substitution (^EE^SlmA) might fail to homodimerize and potentially only form active SlmA dimers when the two variants were co-produced. We tested this hypothesis by producing the charge-swapped SlmA derivatives from two complementary expression constructs integrated at different chromosomal locations (*att*λ and *att*HK022). ^RR^SlmA and ^EE^SlmA accumulated normally when both expression constructs produced the same variant (Figure S1). However, when they were produced individually, the charge-swapped derivatives were unable to suppress the synthetic lethal growth defect of a Δ*slmA* strain depleted of the Min proteins (Figure 8B), indicating that they are defective for nucleoid occlusion. Bacterial two-hybrid (BACTH) analysis based on the reconstitution of adenylate cyclase activity from the fragments T18 and T25 [30], further indicated that the loss of nucleoid occlusion activity was due to the inability of ^RR^SlmA or ^EE^SlmA to self interact (Figure 8B). Also, as expected for derivatives unable to homodimerize, the individual ^RR^SlmA or ^EE^SlmA variants were unable to bind the promoter-embedded SBS in order to repress the P_SBS_::*lacZ* reporter (Figure 8B). Although ^RR^SlmA or ^EE^SlmA were unable to self interact, BACTH analysis demonstrated that the two derivatives effectively interacted with each other (Figure 8B). Moreover, co-production of ^RR^SlmA and ^EE^SlmA in the same strain promoted the effective repression of the P_SBS_::*lacZ* reporter and restored nucleoid occlusion activity to SlmA^−^ Min^−^ cells to rescue their growth defect (Figure 8B). The battery of *in vivo* assays therefore strongly support the conclusion that ^RR^SlmA and ^EE^SlmA must specifically heterodimerize in order to bind DNA and disrupt Z-ring assembly.

To test the functionality of heterodimers in which only one monomer possessed an active FtsZ-interaction site, we used site-directed mutagenesis to generate constructs that produce ^RR^SlmA(FRN) or ^EE^SlmA(FRN) variants with disabled active sites. Importantly, these derivatives behaved identically to the wild-type ^RR^SlmA and ^EE^SlmA variants in the BACTH and P_SBS_::*lacZ* repression assays. ^RR^SlmA fusions only showed an interaction with fusions to ^EE^SlmA variants regardless of the status of their FtsZ-binding site, and as long as complementary pairs of ^RR^SlmA and ^EE^SlmA derivatives were combined, P_SBS_::*lacZ* was repressed (Figure 8B, data not shown). Production of ^RR^SlmA(FRN) with ^EE^SlmA restored growth to the Min^−^ SlmA^−^ strain as did the co-production of ^RR^SlmA with ^EE^SlmA(FRN) (Figure 8B). On the contrary, growth was not restored when ^RR^SlmA(FRN) and ^EE^SlmA(FRN) were co-produced to generate heterodimers in which both monomers possessed defective FtsZ-interaction sites (Figure 8B). We therefore infer that mixed SlmA(FRN)-SlmA(WT) dimers are capable of properly mediating nucleoid occlusion and that only one active FtsZ-interaction interface is required per dimer for proper SlmA function.

## Discussion

In order to identify the “active site” of SlmA, we used a genetic selection and screen combination to identify protein variants that fail to properly regulate FtsZ assembly but retain DNA-binding activity. The amino acid changes found in such variants clustered on the surface of the SlmA structure, and several derivatives with substitutions at these positions were shown to be incapable of interacting with FtsZ or regulating its assembly *in vitro*. We therefore conclude that the SlmA surface region defined by the genetic analysis constitutes an FtsZ-interaction interface essential for SlmA function.

### Mechanism of FtsZ regulation by SlmA

In addition to solving the crystal structure of dimeric SlmA, Schumacher and colleagues have also investigated the nature of the SlmA-FtsZ complex using small-angle X-ray scattering (SAXS) [26]. The resolution of this method is low, and a significant amount of computational modeling is involved in generating potential structures of the complex. It was therefore not possible to determine the precise SlmA-FtsZ interface from this study. However, the SAXS results clearly indicated that stoichiometry of the FtsZ-SlmA complex formed *in vitro* is 1∶1 with two FtsZ molecules capable of binding to one SlmA dimer [26]. Using FtsZ-GFP as well as FtsZ in the SAXS analysis, it was also established that the FtsZ molecules bound to each SlmA monomer were in opposing orientations as expected for proteins bound to a symmetric dimer [26]. This result constitutes the only evidence supporting the model that SlmA disrupts Z-ring assembly by promoting the formation of anti-parallel FtsZ protofilaments. Tonthat et al. (2011) [26] argue that the twisted FtsZ bundles observed by EM in the presence of SlmA-DNA also support this model, but the relative orientation of FtsZ protofilaments in the observed bundles is not clear. Furthermore, given that the nature of FtsZ structures formed in polymerization reactions varies widely depending on the solution conditions and the presence of additives 29,31–35, results from EM analysis need to be interpreted with caution and correlated with the *in vivo* phenotypes of mutant alleles when possible. Indeed, in the original work that identified SlmA, we observed the formation of bundles/ribbons of FtsZ protofilaments in the presence of SlmA [23]. We reasoned that this indicated a direct interaction between SlmA and FtsZ, but a definitive conclusion about the physiological significance of these structures was not possible nor was one given. Subsequently, we presented evidence that SlmA functions as an antagonist of FtsZ polymerization that is activated upon binding to SBS-containing DNA [25]. In support of this mode of action, the nucleoid occlusion defective variant SlmA(R73D) also failed to antagonize FtsZ polymerization *in vitro*. Importantly, however, this derivative still promoted the formation of FtsZ bundles/ribbons in the low KCl buffer used in the original SlmA study [25]. This observation strongly suggests that the formation of FtsZ bundle/ribbon structures is not the relevant physiological activity of SlmA.

The biochemical activities of the SlmA variants identified in this study provide further support for SlmA functioning as an FtsZ polymerization antagonist. The variants with the most significant nucleoid occlusion defects *in vivo* were all found to be defective in antagonizing FtsZ assembly *in vitro*. Thus, there is a tight correlation of our *in vitro* data with *in vivo* observations, supporting the physiological relevance of the biochemical assays. Additionally, the identification of SlmA residues critical for interacting with FtsZ allowed us to test whether or not both FtsZ-interaction interfaces in a SlmA dimer are required for nucleoid occlusion function. We found that obligate heterodimers possessing only one functional FtsZ-interaction site were active in promoting nucleoid occlusion *in vivo*. Our results are therefore not consistent with SlmA working via the formation of anti-parallel FtsZ protofilaments since this mode of action predicts a requirement for two functional FtsZ-interaction sites per dimer.

What remains to be determined is how SlmA promotes FtsZ polymer disassembly and the extent to which multiple SlmA dimers bound to DNA cooperate to deter Z-rings from forming over the nucleoid. Based on the biochemical properties it shares with MinC [36], we previously proposed that SlmA functions analogously and may work by promoting protofilament severing at sites in the polymer where GTP has been hydrolyzed to GDP [25]. While further mechanistic studies are required to test this hypothesis, such a catalytic mode of action in which one SlmA dimer severs multiple FtsZ polymers could explain how a relatively small number of SlmA molecules bound to the SBS-containing sites on the chromosome effectively block Z-ring formation in their vicinity. This mode of action may potentially be further enhanced by the “spreading” of SlmA dimers on the DNA surrounding the SBS sites. In this case, FtsZ polymers could be disrupted at many places along their length by an array of SlmA dimers on DNA. Consistent with possible spreading of SlmA to regions surrounding the SBS sites, relatively large segments of DNA spanning several kilobases around the SBSs were enriched in our anti-SlmA chromosomal immunoprecipitation analysis [25]. There are also many more SlmA molecules in the cell (ca. 500 dimers per nucleoid) than SBSs on the chromosome (24–52 sites/nucleoid) [25], [26].

Thus far, however, we have been unable to detect specific spreading of SlmA on SBS-containing DNA *in vitro*, or *in vivo* using a *lacZ* reporter. No perturbation of *lacZ* expression is observed when SBS sites are placed at locations upstream of the promoter driving the reporter. We therefore suspect that, rather than spreading, specific SlmA-SBS complexes may promote the association of additional SlmA dimers with low-affinity SBSs in the vicinity of the high-affinity site, potentially via dimer-dimer contacts made following looping or folding of the intervening DNA. Further studies investigating the potential higher-order association of SlmA with the chromosome should shed light on this issue and whether or not such associations are critical for proper nucleoid occlusion.

### A potential mechanism for SlmA activation upon DNA binding

**Figure 9:**
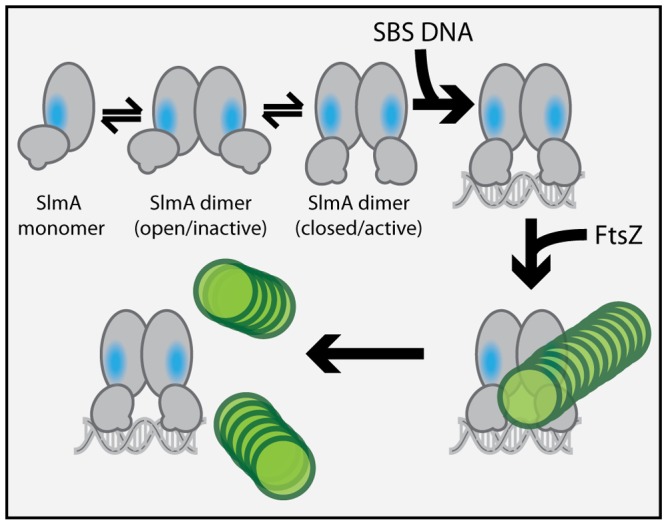
Model for SlmA activation. The SlmA monomer is represented as a two domain structure formed by intersecting ovals. The vertical oval represents the dimerization domain and the angled oval with the small protrusion represents the DNA-binding domain. The small protrusion is the recognition helix, and the blue oval represents the identified FtsZ-interaction interface. The green circles represent FtsZ monomers within a protofilament. At least two conformations of SlmA dimers are envisioned to exist in solution: an open and a closed conformation. The closed conformation is the only one thought to have a fully exposed/functional FtsZ-interaction interface necessary for antagonizing FtsZ polymerization. We propose that in addition to promoting SlmA dimer formation at lower protein concentrations, DNA-binding also stabilizes the closed SlmA conformation thus stimulating its anti-FtsZ activity. See text for a detailed description of the model and the rationale behind it.

We previously observed that in the absence of DNA, high concentrations of SlmA were required to antagonize FtsZ assembly [25]. Since dimer formation also required high concentrations of SlmA, we proposed that dimerization was critical for SlmA activity and that DNA-binding activates SlmA, in part, by promoting dimerization [25]. One possible explanation for this observation was that the FtsZ-interaction site might be formed at the SlmA dimer interface. However, the region of SlmA identified as the FtsZ-interface in our mutational analysis is removed from the region of SlmA-SlmA contact in the structure. Thus, dimerization alone is unlikely to modulate the SlmA-FtsZ interation. A conformational change following SlmA dimerization may also be involved (Figure 9). Interestingly and in support of this hypothesis, the FtsZ-interface we identified maps to a region of the SlmA structure that corresponds to an area that has been shown to be conformationally flexible in other TetR family DNA-binding proteins (Figure 6).

Most of the TetR-like proteins characterized to date are transcriptional repressors that regulate the expression of drug/chemical-resistance pumps [37]. Studies comparing the DNA-bound and drug-bound crystal structures of TetR and QacR indicate that drug-binding likely results in structural changes that promote the disassociation of the repressor proteins from their DNA operator [27], [28]. These structural changes are most significant at the junction of helix-5 and helix-6 and involve either the shortening of helix-6 or the elongation of helix-5 by one turn for TetR and QacR, respectively [27], [28]. In both cases, structural changes at this junction ultimately appear to cause an outward rotational movement of the DNA-binding domains. Such movements are thought to result in induction because the DNA-recognition helices of the repressor dimer move farther apart such that their spacing is no longer optimal for binding to successive major grooves on DNA [27], [28]. Analogous structural alterations have been observed for different crystal forms of AcrR. These forms are believed to represent the DNA-bound and drug-bound conformations of the protein [38], suggesting that TetR proteins are conformationally flexible in the absence of their ligands.

In the structure of the free form of SlmA solved by Schumacher and co-workers [26] the recognition helices (α-3) are 50 Å apart when measuring the distance between the alpha carbons of conserved Y49 residues of each monomer. This distance is greater than the 45 Å spacing for the corresponding residues in drug-bound QacR and much wider than the 34 Å spacing between major grooves in B-DNA. The conformer of SlmA observed in the crystal structure is therefore analogous to the drug-bound conformers of other TetR-like proteins and thus not likely to be competent for DNA-binding. This possibility suggests that in the absence of DNA, SlmA dimers may exist in equilibrium between at least two forms: the one observed in the crystal structure with wide HTH spacing (open conformer), and another where the HTH domains rotate inward to reduce their spacing (closed conformer). We propose that the closed dimer is the active division inhibitor and that DNA-binding promotes its formation to activate SlmA (Figure 9). We envision two possible and nonexclusive mechanisms by which a conformational transition between open and closed states could activate SlmA. Our mutational analysis implicates F65 as being critical for the interaction with FtsZ. In the open conformation, this residue is largely buried behind the DNA-binding domain (Figure 6). Thus, the FtsZ-interface may be partially occluded in this conformation rendering the protein inactive for FtsZ regulation. Like other TetR proteins, however, DNA-binding is likely to be accompanied by the inward rotation of the DNA-binding domain which, in turn, may expose the full FtsZ-binding site and thus activate the protein. Alternatively or additionally, structural changes in the region of the helix 5–6 junction that are expected to accompany the transition between DNA-free and DNA-bound forms of TetR-like proteins [27], [28] may also contribute to the formation of a functional FtsZ-interaction interface when SlmA binds DNA. Importantly, the potential conformational changes must not only promote an interaction with FtsZ, they are also likely needed to properly expose or position residues that are directly involved in breaking down FtsZ protofilaments. While additional structural studies are required to test this model, it is attractive because it raises the possibility that SlmA represents a special subclass of TetR-like proteins that have adapted conformational changes normally associated with inducer binding to modulate its ability to interact with a partner protein. In the case of SlmA, this would ensure that only the DNA-bound form can disrupt FtsZ polymerization. However, given the broad distribution and large numbers of TetR-like proteins produced by bacteria [37], it would not be surprising if other regulatory systems involving TetR-like proteins use a similar strategy to control their interactions with partner proteins in response to DNA binding or release.

## Materials and Methods

### Bacterial strains, plasmids, and growth medium

Bacterial strains and plasmids used in this study are listed in Tables S1 and S2, respectively. A detailed description of the construction procedure for each plasmid is given in Text S1. Cells were grown in LB-1% NaCl [1% tryptone, 0.5% yeast extract, 1.0% NaCl] or minimal M9 medium [39] supplemented with 0.2% casamino acids and 0.2% sugar (glucose, arabinose or maltose as indicated). Unless otherwise indicated, antibiotics were used at 10 (chloramphenicol; Cm, and tetracycline; Tet), 15 (ampicillin; Amp), or 20 (kanamycin; Kan) µg/ml when cells were grown in LB. When cells were grown in M9 medium the concentrations were adjusted to 12.5, 25, 25, or 50 µg/ml for Tet, Cm, Amp, and Kan, respectively.

### Construction of a *lacZ* reporter repressed by SlmA

The chromosomal region of TB10 [40] encompassing the *lacI* gene and *lac* promoter was replaced with a kanamycin resistance cassette and a synthetic promoter containing a SlmA binding sequence (SBS) inserted between the −35 and −10 promoter elements (Kan^R^-P_sbs_) using lambda Red recombineering. The DNA fragment for recombineering was amplified using pHC558 as a template with the primer pairs 5′-CCGGAAGGCGAAGCGGCATGCATTTACGTTGACACCATCGTTGAGCGATTGTGTAGGCTG-3′ and 5′-CAGTGAATCCGTAATCATGGTCATAGCTGTTTCCTGTGTGTATCGTGAGGATGCGTCATC-3′. Following recombineering, the Kan^R^-P_sbs_::*lacZ* reporter was moved by P1 transduction into HC302 [Δ*slmA*] to generate strain HC328 [Δ*slmA* P_sbs_::*lacZ*].

### PCR-based mutagenesis of *slmA* for the selection and screen

The *slmA* gene in pHC583 was mutagenized by PCR amplification with Taq polymerase. The primer pairs 5′-GCTA*TCTAGA*CACATACGCATCCGAATAACG-3′ and 5′-CGTA*AAGCTT*AGAAACTCGCCGGATGAAAAG-3′ were used for the amplification. The resulting mutagenized fragments were digested with XbaI and HindIII, and ligated with identically digested pHC583 so that they replaced the WT copy of *slmA*. The ligations were transformed into electrocompetent DH5α(λpir) cells to yield approximately 50,000 transformants. The transformant colonies were resuspended in LB broth, and plasmid DNA was purified using the Qiaprep plasmid purification kit. The resulting mutagenized plasmid preparation was integrated at the *att*HK022 site in HC328 by using the helper plasmid pTB102 as described previously [41]. Approximately one million colonies resulting from the integration were resuspended in LB. The resuspended cells were then grown for the preparation of electrocompetent cells and transformed with pHC534, a multicopy plasmid containing two tandem SBSs, to make the final library for mutant isolation [HC328(attHKHC583*)/pHC534]. Approximately four million colonies from this final transformation were resuspended in LB for the mutant selection.

### Identification of non-functional SlmA variants

The HC328(attHKHC583*)/pHC534 mutant library was diluted in LB to 10,000 cfu/mL and 100 µL of the dilution was spread on LB plates containing 50 µg/mL ampicillin, 1 mM IPTG, and 40 µg/mL X-gal to select for cells expressing non-functional *slmA* alleles. Most of the survivors formed blue colonies, suggesting that they were producing SlmA variants that were either unstable or defective in DNA binding and therefore unable to repress the P_sbs_::*lacZ* reporter. Survivors forming white colonies, on the other hand, were expected to produce SlmA variants that were defective for FtsZ regulation but functional for DNA binding. White colonies were picked and tested more stringently for potential DNA-binding defects by plating them on LB X-gal plates with a lower IPTG concentration (100 µM) and monitoring color development following two days of growth at 30°C. Mutants that remained white were selected for further analysis. The *slmA* gene from each of the selected isolates was then amplified with primer pairs 5′-GCTATCTAGACACATACGCATCCGAATAACG-3′ and 5′-GACGAAAGTGATTGCGCCTACC-3′ and sequenced using the primer 5′-GACGAAAGTGATTGCGCCTACC-3′ to identify the mutations.

### Characterization of the *slmA* alleles

To further characterize the isolated *slmA* alleles, genes with single point mutations were amplified from the integrated pHC583 construct with the primers 5′-GCTA*TCTAGA*CACATACGCATCCGAATAACG-3′ and 5′-CGTA*AAGCTT*AGAAACTCGCCGGATGAAAAG-3′. The amplified fragments were then used to replace WT *slmA* in pHC531 following digestion with XbaI and HindIII. The resulting plasmids were integrated at *attλ* of strain HC278 [P_ara_::*minCDE* Δ*slmA*]. In this strain, we were able to test the nucleoid occlusion function of the mutants by testing their ability to suppress the lethal Min^−^ SlmA^−^ defect of HC278 when it is grown in the absence of arabinose. Overnight cultures of HC278 cells containing the integrated constructs were grown in M9-arabinose medium. They were normalized for their OD_600_, serially diluted in LB, and 5 µL of each dilution was spotted on LB agar lacking arabinose but containing 1 mM IPTG for induction of the *slmA* alleles. The plates were imaged after incubation for 1 day at 30°C. To confirm the DNA-binding activity of the variants, the constructs were transduced from the HC278 background to HC328 [Δ*slmA* P_sbs_::*lacZ*] and tested for their ability to repress *lacZ* expression. HC328(att*λ*HC531) derivatives were streaked on LB plates containing 100 µM IPTG and 40 µg/mL X-gal. Individual colonies were imaged after incubation for two days at 30°C. To check the localization of the new SlmA variants, the *slmA* alleles were transferred to a gfp-fusion vector where they were under control of the *lac* promoter (see Text S1). The resulting constructs were integrated at the *att*HK022 site of HC259 [Δ*slmA*]. Overnight cultures of the resulting strains were diluted 1∶100 into LB supplemented with 1 mM IPTG and grown to an OD_600_ of 1.0 at 30°C. Cells were visualized on 1.2% agarose pads using DIC and fluorescence optics.

### Site-directed mutagenesis of *slmA*

To identify additional residues important for the anti-FtsZ activity of SlmA, constructs producing variants with the substitutions F65A, S69N, S69A, or R101D were constructed in the pHC531 background using the QuikChange procedure (Stratagene). We also generated a triple mutant allele [*slmA*(FRN)] with all three of the F65A, R73D, and N102S substitutions and obligate heterodimer alleles, *slmA*(E167R) and *slmA*(R175E), using overlap extension PCR. A detailed procedure is described in Text S1.

### Biochemical assays

Protein purifications and the biochemical assays used to characterize the SlmA variants were performed exactly as described previously [25]. The only exception was that the gel shift assays used a fluorescently labeled probe instead of a radiolabeled one. The probe was prepared by PCR using pHC647 as a template and the primers 5′-ACAGGTTTCCCGACTGGAAAG-3′ and 5′-Alex488N-ATGCAGCTCCCGGAGACGGTCAC-3′. After running the gel, the fluorescence probe signal was detected using a Typhoon 9400 imager (GE Healthcare).

## Supporting Information

Figure S1 Accumulation of ^RR^SlmA and ^EE^SlmA derivatives. Overnight cultures of TB57 [P_ara_::*minCDE*], HC278 [P_ara_::*minCDE* Δ*slmA*], and HC278 containing integrated expression plasmids producing the indicated SlmA variant were diluted and grown in LB broth supplemented with 0.5 mM IPTG to an OD_600_ of 0.6. Protein extracts were prepared and proteins in 10 and 20 µg of total extract were separated by SDS-PAGE. SlmA was then detected by immunoblotting with affinity-purified anti-SlmA antibodies. Note that the SlmA(FRN) derivatives run slightly faster than those without the FRN substitutions.
(TIF)

Table S1 Lists strains used in this study.
(DOC)

Table S2 Lists plasmids used in this study.
(DOC)

Text S1 Details for plasmid constructions and other supplementary protocols are given.
(DOC)
