# Supplementary figures and images for: Identification of the SlmA Active Site Responsible for Blocking Bacterial Cytokinetic Ring Assembly over the Chromosome
Source: PLoS Genet. 2013 Feb 14;9(2):e1003304. doi: 10.1371/journal.pgen.1003304 (PMC3573117; doi:10.1371/journal.pgen.1003304)

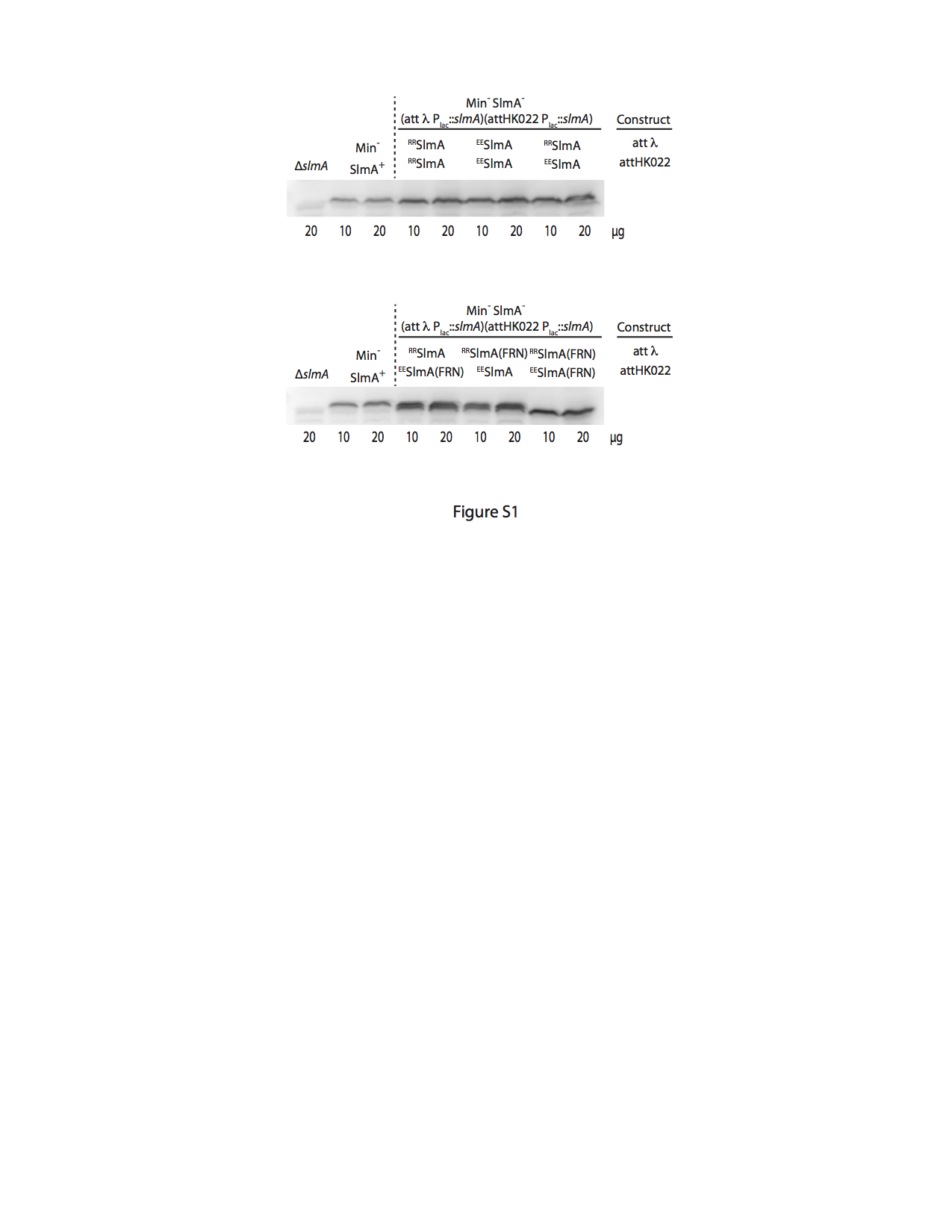

Supplement: Figure S1 — Accumulation of RRSlmA and EESlmA derivatives. Overnight cultures of TB57 [Para::minCDE], HC278 [Para::minCDE ΔslmA], and HC278 containing integrated expression plasmids producing the indicated SlmA variant were diluted and grown in LB broth supplemented with 0.5 mM IPTG to an OD600 of 0.6. Protein extracts were prepared and proteins in 10 and 20 µg of total extract were separated by SDS-PAGE. SlmA was then detected by immunoblotting with affinity-purified anti-SlmA antibodies. Note that the SlmA(FRN) derivatives run slightly faster than those without the FRN substitutions. (TIF) [file pgen.1003304.s001.tif]
