# Supplementary material for: Identification of the SlmA Active Site Responsible for Blocking Bacterial Cytokinetic Ring Assembly over the Chromosome
Source: PLoS Genet. 2013 Feb 14;9(2):e1003304. doi: 10.1371/journal.pgen.1003304 (PMC3573117; doi:10.1371/journal.pgen.1003304)
Supplement: Table S1 — Lists strains used in this study. (DOC) [file pgen.1003304.s002.doc]

**Table S1.** Bacterial strains used in this study.

| Strain | Genotypea | Source/Referenceb |
| --- | --- | --- |
| DH5α | F– *hsdR*17 *deoR recA*1 *endA*1 *phoA supE*44 *thi*-1 *gyrA*96 *relA*1 Δ(*lacZYA-argF*)U169 ϕ80d*lacZ*ΔM15 | Gibco BRL |
| HC258(λDE3) | *ompT* rB– mB– Δ*slmA*::KanR(PlacUV5::T7*gene1*) | This study |
| Rosetta(λDE3) | *ompT* rB- mB- *galdcm* (PlacUV5::T7*gene1*) | Novagen |
| MG1655 | *rph*1 *ilvGrfb*-50 | [1] |
| BW25113 | Δ(*araD*-*araB*)567 Δ*lacZ*4787(::*rrnB*-3) *rph*-1 Δ(*rhaD*-*rhaB*)568 *hsdR*514 | [2] |
| JW5641-1 | BW25113 Δ*slmA*::KanR | [2] |
| TB10 | *rph*1 *ilvGrfb*-50 λΔ*cro-bionad*::Tn10 | [3] |
| TB28 | MG1655 Δ*lacIZYA*::*frt* | [4] |
| TB57 | TB28 (*frt araC* Para)::*minCDE* | [4] |
| HC259 | TB28 Δ*slmA*<>frt | [5] |
| HC260 | TB10 *zapA*-*gfp* CamR | [5] |
| HC277 | MG1655 Δ*slmA*::KanR | [5] |
| HC278 | TB57 Δ*slmA*<>*frt* | [5] |
| HC286 | TB28 *zapA*-*gfp* *frt* | P1(HC260) x TB28/pCP20 |
| HC290 | TB28 *zapA*-*gfp* *frt* Δ*slmA*::KanR | P1(JW5641-1) x HC286 |
| HC302 | MG1655 Δ*slmA*<>*frt* | HC277/pCP20 |
| HC324 | TB10 KanR Psbs::*lacZ* | recombineering |
| HC328 | MG1655 Δ*slmA*<>*frt* KanR Psbs::*lacZ* | P1(HC324) x MG1655 |

a The KanR and CamR cassette are flanked by *frt* sites for removal by FLP recombinase. An *frt* scar remains following removal of the cassette using FLP expressed from pCP20.

b Strain constructions by P1 transduction are described using the shorthand: P1(donor) x recipient. Strains resulting from the removal of frt-flanked antibiotic resistance gene using pCP20 are indicated as: Parental strain/pCP20.
