# Supplementary material for: Identification of the SlmA Active Site Responsible for Blocking Bacterial Cytokinetic Ring Assembly over the Chromosome
Source: PLoS Genet. 2013 Feb 14;9(2):e1003304. doi: 10.1371/journal.pgen.1003304 (PMC3573117; doi:10.1371/journal.pgen.1003304)
Supplement: Table S2 — Lists plasmids used in this study. (DOC) [file pgen.1003304.s003.doc]

**Table S2.** Plasmids used in this study.

| Plasmid | Genotypea | ori | Source/Reference |
| --- | --- | --- | --- |
| pTB183 | *attHK022 bla lacIq* Plac::*gfp-zapA* | R6K | [6] |
| pHC482 | *attHK022 bla lacIq* Plac::*gfp-slmA(R73D)* | R6K | [5] |
| pHC505 | *attHK022 bla lacIq* Plac::*gfp-slmA(T33A)* | R6K | [5] |
| pHC515 | *bla* | pBR/colE1 (pUC) | [5] |
| pHC531 | *attλ cat lacIq* Plac-m3::*slmA* | R6K | [5] |
| pHC529 | *bla lacIq* PT7::*h-sumo-ftsZ(D212N)* | pBR/colE1 | [5] |
| pHC534 | *bla 2X SBS* | pBR/colE1 (pUC) | [5] |
| pHC558 | KanR-Psbs | R6K | This study |
| pHC583 | *attHK tetA lacIq* Plac-m3::*slmA* | R6K | This study |
| pHC610-614  pHC678-679  pHC720 | *attλ cat lacIq* Plac-m3::*slmA(N102S), slmA(F65I), slmA(G97D), slmA(L105Q), slmA(L94Q), slmA(F65A), slmA(R101D), slmA(F65A/R73D/N102S)* | R6K | This study |
| pHC625  pHC627-631  pHC684-685 | *attHK022 bla lacIq* Plac::*gfp-slmA*  *-slmA(N102S), -slmA(F65I), -slmA(G97D), -slmA(L105Q), -slmA(L94Q), -slmA(F65A), -slmA(R101D)* | R6K | This study |
| pHC652  pHC694-695  pHC722 | *bla lacIq* PT7::*h-sumo-slmA(N102S), -slmA(F65A), -slmA(R101D), -slmA(F65A/R73D/N102S)* | pBR/colE1 | This study |
| pHC746-747  pHC752 | *attλ cat lacIq* Plac-m3::*slmA(E167R), slmA(R175E),* or *slmA(F65A/R73D/N102S/E167R)* | R6K | This study |
| pHC748-749  pHC754 | *attHK tetA lacIq* Plac-m3::*slmA (E167R), slmA(R175E),* or *slmA(F65A/R73D/N102S/R175E)* | R6K | This study |

a PT7, PR, Plac, and Para indicate the phage T7, λR, lactose, and arabinose promoters, respectively. Plac-m3 is a *lac* promoter derivative with TATATT as its -10 element. Numbers in parenthesis indicate the codons included in the relevant clones.
