# Supplementary material for: Identification of the SlmA Active Site Responsible for Blocking Bacterial Cytokinetic Ring Assembly over the Chromosome
Source: PLoS Genet. 2013 Feb 14;9(2):e1003304. doi: 10.1371/journal.pgen.1003304 (PMC3573117; doi:10.1371/journal.pgen.1003304)
Supplement: Text S1 — Details for plasmid constructions and other supplementary protocols are given. (DOC) [file pgen.1003304.s004.doc]

**Supporting Material For:**

**Identification of the SlmA active site responsible for blocking bacterial cytokinetic ring assembly over the chromosome**

Hongbaek Cho and Thomas G. Bernhardt*

Department of Microbiology and Immunobiology

Harvard Medical School

Boston, MA 02115

*To whom correspondence should be addressed.

Thomas G. Bernhardt, Ph.D.

Harvard Medical School

Department of Microbiology and Immunobiology

HIM Building, Room 1026

4 Blackfan Circle

Boston, Massachusetts 02115

Phone: (617) 432-6971

Fax: (617) 738-7664

e-mail: [thomas_bernhardt@hms.harvard.edu](mailto:thomas_bernhardt@hms.harvard.edu)

**MATERIALS AND METHODS**

**Bacterial strains, and plasmids.**

Bacterial strains and plasmids used in this study are listed in Tables S1 and S2, respectively. A detailed procedure for each plasmid construction is given below. Restriction sites encoded in primers used in plasmid constructions are italicized and underlined in the primer sequences given in the description. In the oligonucleotides used for site-directed mutagenesis, the mutated bases are highlighted in bold.

**pHC558**

To make a PCR template for amplifying the synthetic P_sbs_ promoter linked to a Kan^R^ cassettte (Kan^R^-P_sbs_) for recombineering, oligonucleotides 5’-*TCGAC***TTGAGT**AAGTGAGCGCTCACTTAC**TATAAT**GTGTG*G*-3’ and 5’-*AATTC*CACAC**ATTATA**GTAAGTGAGCGCTCACTT**ACTCAA***G* -3’ were annealed and ligated with pTB29 digested with SalI and EcoRI to generate pHC558.

**pHC583**

For pHC583 [*attHK022 tetA lacI^q^* P*_lac-m3_*::*slmA*], a DNA fragment containing *lacI^q^*, the P_lac-m3_ promoter, a *slmA* fragment containing the coding sequence along with 106 bp of upstream sequence and 36 bp of downstream sequence was liberated from pHC531 [5] with BglII and HindIII and ligated with pDY75 [7] digested with the same enzymes.

**pHC610-614**

The *slmA* insert in pHC531 [*attλ cat lacI^q^* P*_lac-m3_*::*slmA*] [5] was replaced with mutant alleles to test their ability to correct the synthetic lethal phenotype of Min^-^ SlmA^-^ cells. Mutant *slmA* alleles (N102S, F65I, G97D, L105Q, or L94Q) were amplified from colonies of the isolated mutant strains with the primers, 5’-GCTA*TCTAGA*CACATACGCATCCGAATAACG-3’ and 5’-GACGAAAGTGATTGCGCCTACC-3’. The amplified DNA was digested with XbaI and HindIII and ligated with pHC531 digested with the same enzymes.

**pHC678-679**

The *slmA* insert in pHC531 was mutated to encode SlmA(F65A) or SlmA(R101D) using the QuikChange procedure (Stratagene), with the mutagenic primer 5’-AGTAATCAGGCTATCTTCGAT**CGC**CTCAATCAGGCTATCGAACAT-3’ for F65A and 5’-GATGCGGGTCAGGCCAGGATT**ATC**CTCACCAAAACCGAGAAGCAG-3’ for R101D.

**pHC720**

The triple mutant *slmA*(F65A, R73D, N102S) was synthesized by overlap extension PCR. The first fragment containing the F65A and R73D substitutions was amplified with the primers 5’-GCTATCTAGACACATACGCATCCGAATAACG-3’ and 5’-CAGGTTGATG**TC**AGTAATCAGGCTATCTTCG-3’ using pHC678 as a template. The second fragment containing the R73D and N102S substitutions was amplified with the primers 5’-AGCCTGATTACT**GA**CATCAACCTGATTCTGAAAG-3’ and 5’-GACGAAAGTGATTGCGCCTACC-3’ using pHC610 as a template. The two PCR products were then mixed together with the primer pair 5’-GCTATCTAGACACATACGCATCCGAATAACG-3’ and 5’-GACGAAAGTGATTGCGCCTACC-3’ to make the full length *slmA* overlap extension product containing F65A, R73D, and N102S mutations. The overlap extension product was digested with XbaI and HindIII and ligated to pHC531 digested with the same enzymes.

**pHC625, pHC627-631, and pHC684-685**

The *slmA* mutant alleles (WT, N102S, F65I, G97D, L105Q, L94Q, F65A, or R101D) were amplified with the primer pairs 5’-GCT*GGATCC*GCAGAAAAACAAACTGCGAAAAG-3’ and 5’-GCAT*AAGCTT*TTACTGCAACTGTGCCGCAAT-3’ using pHC531, pHC610-614, and pHC684-685 as templates, respectively. The amplified fragments were digested with BamHI and HindIII and ligated with pTB183 digested with the same enzymes to generate the CRIM plasmids [*attHK022 bla lacI^q^* P*_lac_*::*gfp-slmA(mutant)*].

**pHC652, pHC694-695, pHC722**

The *slmA* mutant alleles (N102S, F65A, R101D, or F65A/R73D/N102S) were amplified with 5’-GGTGGTT*GCTCTTC*CGGTGCAGAAAAACAAACTGCGAAAAGG-3’ and 5’-GCCT*CTCGAG*TTACTGCAACTGTGCCGCAATTAGC-3’ using pHC610, pHC678-679, and pHC720 as templates, respectively. The amplified fragments were digested with SapI/XhoI and ligated to pTB146 [8] digested with the same enzymes to make plasmids expressing His6-SUMO-SlmA (N102S, F65A, R101D, or F65A/R73D/N102S) for the purification of untagged SlmA mutant proteins.

**pHC746-749**

*slmA*(E167R) and *slmA*(R175E) alleles were generated by overlap extension with primers introducing E167R or R175E mutation. The 5’ end of *slmA* was amplified with 5’-GCTATCTAGACACATACGCATCCGAATAACG-3’ and 5’-CAGCATACCGCGACAGAAGGCCAGGATCTGG-3’ (for E167R) or 5’-AAATTCGCTTTCGACAAAACGTGACAGCATACC-3’ (for R175E) using pHC531 as a template. The 3’ end of slmA was amplified with 5’-GCCTTCTGTCGCGGTATGCTGTCACGTTTTGTC-3’ (for E167R) or 5’-CGTTTTGTCGAAAGCGAATTTAAATACCGC-3’ (for R175E) and 5’-GACGAAAGTGATTGCGCCTACC-3’ using pHC531 as a template. The two PCR products were then extended and amplified with the primer pair 5’-GCTATCTAGACACATACGCATCCGAATAACG-3’ and 5’-GACGAAAGTGATTGCGCCTACC-3’ to make overlap extension products, *slmA*(E167R) or *slmA*(R175E). The overlap extension products were digested with XbaI and HindIII and ligated to pHC531 (for pHC746-747) or pHC583 (for pHC748-749) digested with the same enzymes.

**pHC752 and pHC754**

*slmA*(F65A/R73D/N102S/E167R) allele for pHC752 and *slmA*(F65A/R73D/N102S/R175E) allele for pHC754 were generated in the same way as *slmA*(E167R) and *slmA*(R175E), except for using pHC720 as a template to amplify 5’ end of *slmA*(F65A/R73D/N102S). These alleles were digested with XbaI and HindIII and ligated with pHC531 (for pHC752) or pHC583 (for pHC754) digested with the same enzymes.

**REFERENCES**

1. Guyer MS, Reed RR, Steitz JA, Low KB (1981) Identification of a sex-factor-affinity site in E. coli as gamma delta. Cold Spring Harb Symp Quant Biol 45 Pt 1: 135–140.

2. Baba T, Ara T, Hasegawa M, Takai Y, Okumura Y, et al. (2006) Construction of Escherichia coli K-12 in-frame, single-gene knockout mutants: the Keio collection. Mol Syst Biol 2: 2006.0008. doi:10.1038/msb4100050.

3. Johnson JE, Lackner LL, Hale CA, de Boer PAJ (2004) ZipA is required for targeting of DMinC/DicB, but not DMinC/MinD, complexes to septal ring assemblies in Escherichia coli. J Bacteriol 186: 2418–2429.

4. Bernhardt TG, de Boer PAJ (2004) Screening for synthetic lethal mutants in Escherichia coli and identification of EnvC (YibP) as a periplasmic septal ring factor with murein hydrolase activity. Mol Microbiol 52: 1255–1269. doi:10.1111/j.1365-2958.2004.04063.x.

5. Cho H, McManus HR, Dove SL, Bernhardt TG (2011) Nucleoid occlusion factor SlmA is a DNA-activated FtsZ polymerization antagonist. Proc Natl Acad Sci USA 108: 3773–3778. doi:10.1073/pnas.1018674108.

6. Bendezú FO, de Boer PAJ (2008) Conditional lethality, division defects, membrane involution, and endocytosis in mre and mrd shape mutants of Escherichia coli. J Bacteriol 190: 1792–1811. doi:10.1128/JB.01322-07.

7. Yang DC, Tan K, Joachimiak A, Bernhardt TG (2012) A conformational switch controls cell wall-remodelling enzymes required for bacterial cell division. Mol Microbiol 85: 768–781. doi:10.1111/j.1365-2958.2012.08138.x.

8. Bendezú FO, Hale CA, Bernhardt TG, de Boer PAJ (2009) RodZ (YfgA) is required for proper assembly of the MreB actin cytoskeleton and cell shape in E. coli. EMBO J 28: 193–204. doi:10.1038/emboj.2008.264.
